# The Home, Block, and Community Environments and Biomarkers of Aging in the National Health and Aging Trends Study

**DOI:** 10.1093/geroni/igaa057.1976

**Published:** 2020-12-16

**Authors:** Laken Roberts, Laura Samuel, Danielle Boyce, Melissa Hladek, Sarah LaFave, Sarah Szanton

**Affiliations:** 1 Johns Hopkins University School of Nursing, Baltimore, Maryland, United States; 2 Johns Hopkins University, Baltimore, Maryland, United States

## Abstract

Prior studies have linked household and community conditions to the health and functioning of older adults. However, few studies have investigated associations between household, block, and community environmental conditions with biomarkers of aging. This study used NHATS Round 7 (2017) data on 3,283 community-dwelling older adults to test cross-sectional associations between interior and exterior household disorder, block disorder, community social cohesion, and four biomarkers: C-reactive protein, hemoglobin A1c, cytomegalovirus, and interleukin-6. Survey-weighted models adjusted for age, sex, race/ethnicity, income, education, homeownership, housing type, and metropolitan area; HbA1c was stratified by diabetes diagnosis. Greater interior household disorder was associated with higher IL-6 (β=0.06, SE=0.025, p=0.014) and, among diabetics, greater block disorder was associated with higher HbA1c (β=0.11, SE=0.05, p=0.046). These results link home and block environmental characteristics with biomarkers of aging, suggesting that modifiable aspects of older adults’ living environments may be related to disease and disability risk via physiologic dysregulation.

